# Leadership style, conflict management behavior, and self-regulation among Uzbek managers: differential prediction by intrinsic and extrinsic religious orientation in a post-Soviet Muslim-majority workforce

**DOI:** 10.3389/fpsyg.2026.1890044

**Published:** 2026-08-28

**Authors:** Jakhongir M. Ibragimov

**Affiliations:** Department of Psychology of Religion and Pedagogy, International Islamic Academy of Uzbekistan, Tashkent, Uzbekistan

**Keywords:** Central-Asia, conflict management, cross-cultural management, individual differences, leadership style, organizational behavior, religious orientation, Uzbekistan

## Abstract

**Background:**

Religious orientation is an understudied individual difference predictor of managerial behavior in organizational psychology with particular application to contexts outside of Western Christianity. Uzbekistan, a unique setting where a sustained post-Soviet Islamic revival and rapid professionalization of the workforce converge, is valuable for examining whether Allport's intrinsic-extrinsic religiosity distinction predicts leadership style, workplace conflict handling, and self-regulation among practicing managers.

**Methods:**

A cross-sectional correlational study was conducted with an anonymous online convenience sample of 286 managers and supervisors in educational, private and state organizations. The participants filled in the Santa Clara Strength of Religious Faith Questionnaire (SCSORF), the Shcherbatykh Individual Structure of Religiosity Scale, the Ilin Management Style Inventory (abridged 33-item version), the Nikiforov integral self-control scale, and the Thomas-Kilmann Conflict Mode Instrument. Analysis was by bivariate correlation, One-way ANOVA, and multivariate regression with demographic covariates.

**Results:**

Religious belief was positively related to accommodating conflict style (*r* = +0.17, *p* = 0.004) but this bivariate association was not significant after multivariate adjustment for age which was the most dominant predictor (β = 0.24, *p* = 0.004). At the dimensional level, the facets of intrinsic religiosity (inner need, recognition of a Creator) were the main correlates of accommodation, whereas extrinsic religiosity (attention to outward religious markers) exhibited only a marginal, non-significant trend toward authoritarian leadership style (β = 0.16, *p* = 0.051). Religious belief was not directly linked to democratic or liberal leadership styles or to integral self-control. In general, the demographic variables (age, gender) were better predictors than was religiosity.

**Conclusion:**

The findings provide the first empirical evidence in a Central Asian Muslim managerial sample that Allport's intrinsic—extrinsic distinction yields differentiated organizational behavioral correlates: intrinsic orientation correlates with conflict-handling accommodation; extrinsic orientation shows a non-significant trend toward authoritarian leadership tendencies that deserve replication. The findings add to cross-cultural organizational psychology by showing that religious orientation is a significant individual-difference predictor of workplace behavior in a post-Soviet Muslim-majority context with practical implications for leadership development and conflict management training.

## 1 Introduction

Individual differences in values, beliefs and motivation fundamentally predict managerial behavior (Judge and Piccolo, 2004; Walumbwa et al., 2008), but one of the most pervasive sources of human values, religious orientation, is a relatively understudied predictor of workplace behavior in organizational psychology, particularly outside of the North American and Western European contexts. This is an important gap to fill. Religion shapes moral reasoning, norms for interpersonal interaction, and self-regulatory strategies in ways that organizational theory suggests will influence leaders' decision-making, conflict management, and self-regulation (McCullough and Willoughby, 2009; Fry, 2003). Allport and Ross (1967) intrinsic-extrinsic distinction, the most theoretically rigorous approach to linking religion to behavior, suggests that the motivational function of religion, internalized as a master motive or used instrumentally for social ends, predicts behavioral outcomes more precisely than the mere fact of religious belief. This framework, which has been developed mainly with samples of North American Christians, has been rigorously tested in the context of religious values, and the organizational contexts are substantively different from Western prototypes, leaving the cross-cultural generalizability an open empirical question (Saroglou, 2011; Saroglou et al., 2004).

The Republic of Uzbekistan offers a very valuable organizational setting for this study. Since its independence in 1991, the post-Soviet country has experienced a sustained Islamic revival after a period of state-imposed secularism during the Soviet era, in which the state was actively involved in shaping religious life (Khalid, 2007). At the same time, it has accelerated the development of a professional management class through private-sector growth and public administration reform. Thus, Uzbek managers find themselves at the nexus of growing religious commitment and the rapid institutionalization of modern organizational roles to make daily decisions about how to lead subordinates, manage interpersonal conflict, and regulate their own conduct. It is not quantitatively investigated whether the value architecture that Islamic religious orientation provides—and especially the motivational quality of that orientation (intrinsic-extrinsic)—influences these organizational behaviors, yet this constitutes an important gap in cross-cultural organizational psychology.

The confluence of religion and management has been examined in international scholarship under a variety of rubrics. Within the spiritual leadership theory, values, calling, and membership are proximal predictors of leader effectiveness (Fry, 2003; Fry et al., 2017; Yang and Fry, 2018). All three—servant leadership (Greenleaf, 1977; Sun et al., 2019), ethical leadership (Brown et al., 2005), and authentic leadership (Avolio and Gardner, 2005; Walumbwa et al., 2008)—have a moral or values-based component that is in principle compatible with religious motivation. Religiosity-related antecedents of management behavior have been explored in empirical studies in Pakistani higher education (Abbas et al., 2023), Chinese industry (Chen et al., 2012), Malaysian and Indonesian contexts (Jamaludin et al., 2011), Jordanian organizations (Caputo, 2018), and Australian Christian-Muslim comparisons (Wilson and Power, 2004). Recent research has examined the moderating role of religiosity on ethical and pro-organizational behavior in Muslim-majority workplaces (Alqhaiwi et al., 2023; Sulaiman et al., 2022) and the role of spirituality and religiosity in ethical judgment in organizations (Alshehri et al., 2021). What is largely absent, and what forms the motivation for this present study, is rigorous quantitative work in the Central Asian, post-Soviet, Hanafi-Sunni context that has been studied extensively in religious studies but only sparsely in the psychology of religion.

A second theoretical motivation is the well-established, but still-contested, literature on religion and self-regulation. McCullough and Willoughby's (2009) influential meta-analysis found that religious engagement is associated with stronger self-control, but effects are modest and culturally variable (Geyer and Baumeister, 2005). Self-regulation has also been associated with conflict management and interpersonal effectiveness in the work setting (Baumeister and Vohs, 2007; Clinton et al., 2020; Tangney et al., 2004). One of the gaps that the present article seeks to address is whether religious belief operates through self-regulatory mechanisms to affect managerial conduct in a non-Western Muslim sample.

The purpose of this study is three-fold. First, to descriptively estimate the prevalence and structure of religiosity, management styles, self-regulation, and conflict-handling preferences in a convenience sample of 286 Uzbek managers. Second, to test religious beliefs and dimensions of religiosity, measured globally (SCSORF) and dimensionally (Shcherbatykh's eight-factor structure), as predictors of managerial behavior. Third, to compare the predictive validity of inner-oriented religiosity (Inner Need, Recognition of a Creator) and outer-oriented religiosity (attention to external religious markers) and to determine whether there is empirical support for Allport's intrinsic-extrinsic distinction in this context. The main hypotheses are formulated below.

- **H1**. Religious beliefs are positively correlated with democratic management style and negatively correlated with authoritarian management style.
- **H2**. Positive relationship between religious belief and self-regulation.
- **H3**. Religious belief is positively correlated with cooperative conflict resolution styles (cooperation, accommodation) and negatively correlated with competition.

For the purposes of H4, positive managerial behaviors are operationally defined as the set of outcomes that the organizational links to favorable subordinate and team consequences: democratic style, cooperative conflict-resolution styles (collaborating and accommodating), and higher self-regulation (Eagly et al., 2003; Judge and Piccolo, 2004; Tangney et al., 2004).

- **H4**. In keeping with Allport's framework, intrinsic religiosity (inner need, recognition of a creator) is more strongly associated with positive managerial behaviors than extrinsic religiosity (attention to external religious markers).
- **H5**. Women are more religious than men and prefer a more cooperative and accommodating style of conflict.

## 2 Literature review and theoretical framework

### 2.1 Management styles: the classical triad and its variations

The classical typology of management styles—authoritarian, democratic, and laissez-faire (liberal)—is based on the experimental studies of Lewin et al. (1939) and forms the basic scheme on which most subsequent refinements are based. Later work distinguished task-oriented from relationship-oriented leadership (Blake and Mouton, 1964); contingency models related leader effectiveness to the situation (Fiedler, 1967; Hersey and Blanchard, 1969). The transformational-transactional distinction (Bass and Avolio, 1994; Burns, 1978) and the values-laden frameworks of authentic, ethical, and servant leadership (Avolio and Gardner, 2005; Brown et al., 2005; Greenleaf, 1977; Walumbwa et al., 2008) moved the literature toward integrative models of leader behavior that explicitly include moral and motivational elements.

Because the present study measures the classical triad, its three constructs are defined here as they are operationalized by the management-style inventory used here (Agrashenkov, 1997; Il'in, 2008), following the original Lewin et al. (1939) conceptualization. The authoritarian (autocratic) management style is defined as a pattern of centralized, unilateral decision-making in which the manager issues directives, closely monitors execution, and reserves evaluative authority to himself or herself. A democratic management style is defined as a pattern of shared decision-making in which the manager consults subordinates, delegates meaningful authority, and treats subordinates' initiative as a resource. The liberal (laissez-faire) management style is defined as minimal managerial intervention, with decisions and control largely ceded to subordinates or to circumstance. These definitions align item-for-item with the three Ilin subscales described in Section 3.3.2, ensuring correspondence between the constructs as defined and as measured.

Meta-analytic evidence consistently demonstrates that democratic and transformational styles are linked with higher subordinate satisfaction and performance (Eagly et al., 2003; Judge and Piccolo, 2004). Gender differences in style have been well documented and are particularly pertinent to the present study: women tend to be somewhat more transformational and democratic than men, while men tend to be more laissez-faire (Eagly and Carli, 2003; Eagly et al., 1995).

### 2.2 The Allport framework: religious orientation of intrinsic and extrinsic

Allport (1950) in The Individual and His Religion distinguished between religion as the master of personality (intrinsic) and religion as an instrumental means to social belonging, comfort, or status (extrinsic). Allport and Ross (1967) further refined the construct and provided a psychometric measure that has resulted in thousands of empirical studies. The framework has been elaborated in a number of forms, including into “quest” religiosity and into Saroglou's (2011) four-dimensional model of believing, bonding, behaving, and belonging, but the core insight that how a person is religious is more important than whether has remained robust.

Following Allport and Ross (1967), intrinsic religious orientation is defined as religiousness that functions as a master motive: faith is internalized, pursued as an end in itself, and organizes other life domains around it. Extrinsic religious orientation is defined as religiousness used instrumentally—as a means to security, sociability, status, or self-justification. The two orientations are not opposite poles of one dimension but distinct motivational stances toward the same belief content (Allport and Ross, 1967; Hill and Pargament, 2003; Pargament, 1997).

The conceptual hierarchy among the religiosity constructs in this study is therefore as follows. Religious belief refers to the global strength of religious faith, measured by the SCSORF total score, and is neutral with respect to motivational dimensions within religiosity, indexed here by facets of the Shcherbatykh scale (Myagkov et al., 1996): inner religious need (a felt inner necessity of faith independent of external circumstances) and recognition of a Creator (an internalized conviction in a creating God) serve as markers of intrinsic orientation, whereas attention to external religious markers (orientation toward the outward, visible attributes of religious observance) serves as a marker of extrinsic orientation. This mapping follows the content correspondence between Shcherbatykh's facet definitions and the Allport-Ross conceptualization, and it is the basis on which H4 is formulated and tested.

Importantly, the framework makes different behavioral predictions. Intrinsic religiosity, as the integration of beliefs into the core of self, is hypothesized to be related to prosociality, forgiveness, self-control, and moral consistency (Hood et al., 2018; McCullough and Willoughby, 2009; Saroglou et al., 2004). Conversely, extrinsic religiosity has been conceptually linked to conformity, conservatism, and, in some studies, prejudice and authoritarian attitudes (Hill and Pargament, 2003; Saroglou, 2011). The model has been extensively applied to Christian samples, but as Saroglou (2011) has claimed, it needs to be validated in non-Western contexts.

### 2.3 Religion and self-control

Self-regulation is defined as the capacity to override or alter one's dominant responses and to align behavior with standards and long-term goals (Baumeister and Vohs, 2007; Tangney et al., 2004); in this study it is operationalized as integral self-control across emotional, performance, and social domains, as measured by the Nikiforov self-control questionnaire (Nikiforov, 1989; Section 3.3.3). The meta-analysis by McCullough and Willoughby (2009) remains the benchmark of the religion-self-regulation literature. Their conclusion is nuanced: religious engagement is consistently, but modestly, associated with greater self-control, with effects typically in the *r* = 0.10–0.25 range. Mechanisms include the internalization of moral standards, the salience of supernatural monitoring, and self-disciplinary rituals. Self-regulation has been linked to conflict management styles, ethical behavior, and lower burnout in organizational settings (Baumeister and Vohs, 2007; Clinton et al., 2020; Tangney et al., 2004). To the best of our knowledge, these patterns have not been tested in Muslim-majority post-Soviet settings.

### 2.4 Styles of handling conflict

The five strategies generated by Thomas and Kilmann's (1974) two-dimensional model of conflict handling (assertiveness × cooperativeness)—competing, collaborating, compromising, avoiding, and accommodating—have become the workhorse of conflict management research. In this model, competing is high assertiveness with low cooperativeness (pursuing one's own concerns at the other's expense); collaborating is high assertiveness with high cooperativeness (seeking solutions that fully satisfies both parties); compromising is intermediate on both dimensions (mutual partial concession); avoiding is low on both (sidestepping the conflict); and accommodating is low assertiveness with high cooperativeness (yielding one's own concerns to satisfy the other) (Kilmann and Thomas, 1977; Thomas and Kilmann, 1974). Cooperative conflict-resolution styles, as the term is used in H3, denotes the high-cooperativeness modes: collaborating and accommodating. Previous cross-cultural research has consistently shown that individualistic cultures prefer competition and collaboration, while collectivistic cultures prefer accommodation and avoidance (Cai and Fink, 2002; Holt and DeVore, 2005; Ohbuchi et al., 1999; Trubisky et al., 1991). Religion has been theorized as a cultural carrier of values shaping conflict orientation: traditions that emphasize sabr (patience), tawakkul (trust in God), and forbearance—like Islamic ethical thought—should predict accommodating rather than competitive responses (Caputo, 2018; Wilson and Power, 2004).

### 2.5 Uzbekistan the scene

The dominant Hanafi-Sunni tradition in Uzbekistan has a wealth of advisory and management literature, such as the Siyasatnama of Nizam al-Mulk and the Nasihat al-Muluk of al-Ghazali, which historically influenced administrative practice throughout the Persianate world (Idriz, 2020). Since 1991, the state has played an active role in shaping the post-Soviet revival of religious practice. This includes the founding of the International Islamic Academy of Uzbekistan in 1999 (known until 2018 as Tashkent Islamic University), as well as Tashkent Islamic Institute, which has operated since 1971 and provided Islamic education even during the Soviet era. Simultaneously, a generational change in the labor market, the rapid development of the private sector, and the ongoing reform of public administration have produced a population of managers whose value commitments are formed at the intersection of religious revival and global managerial modernity. This makes Uzbekistan a substantive test for the cross-cultural validity of the Allport framework.

Taken together the literature motivates the hypotheses of the present paper. H1 and the leadership component of H5 are grounded in the management-styles literature (Section 2.1) and gender–leadership findings; H4 and the differentiated intrinsic/extrinsic predictions are based on the Allport framework (Section 2.2); H2 is based on the religion–self-regulation literature (Section 2.3); and H3 and the conflict component of H5 are based on the conflict-handling and cross-cultural literature (Section 2.4) and the Uzbek Hanafi–Sunni context (Section 2.5). The study thus examines a set of theoretically derived predictions linking each reviewed construct to a particular managerial outcome.

### 2.6 Conceptual framework

**Figure 1:**
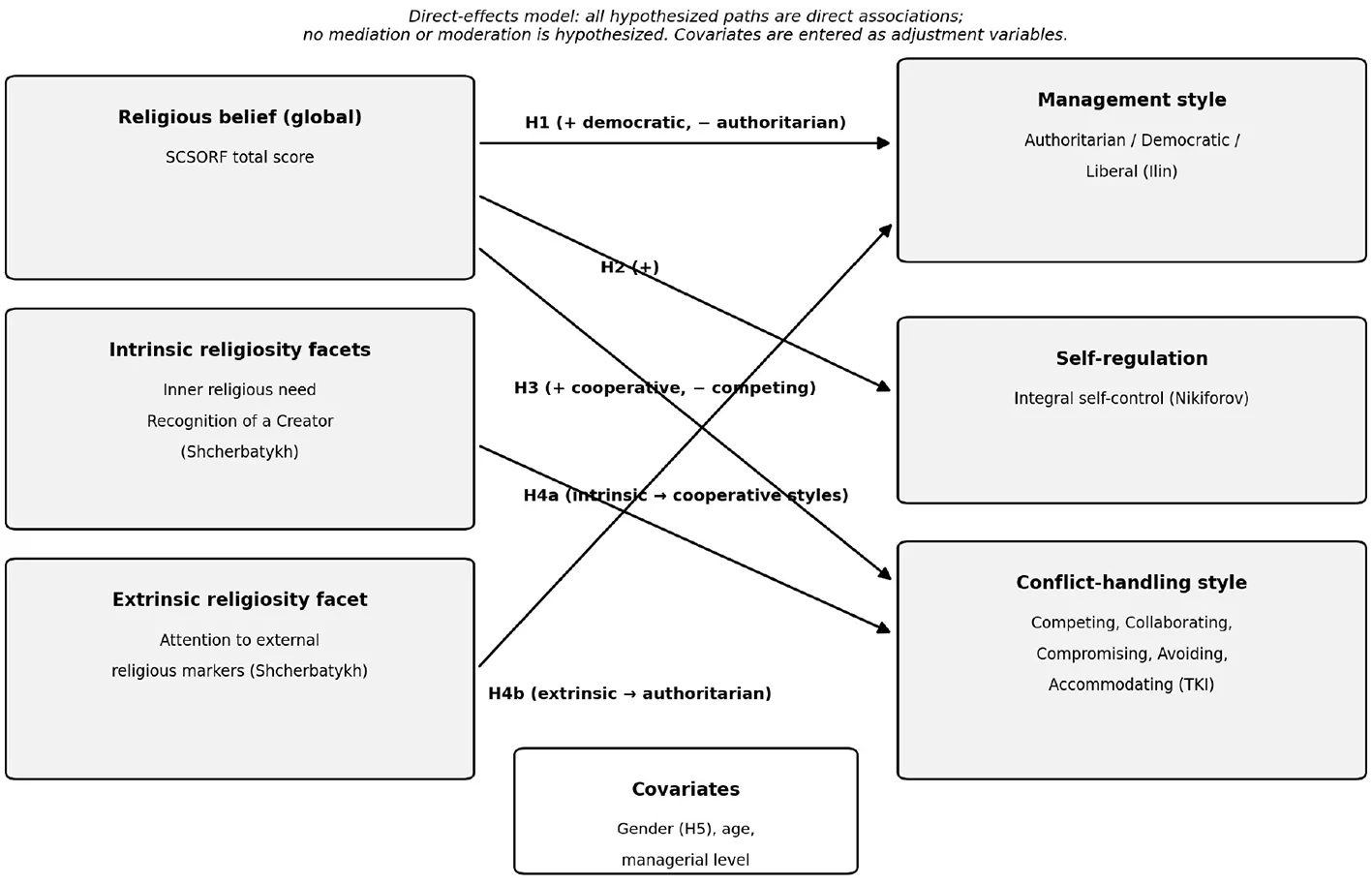
Conceptual framework of the study. All paths represent hypothesized direct associations; no mediation or moderation is hypothesized. Gender, age, and managerial level are entered as adjustment covariates.

Figure 1 summarizes research model. The model consists solely of direct associations: the global religious belief index (SCSORF) is hypothesized to relate directly to management style (H1), self-regulation (H2), and conflict-handling style (H3); the intrinsic facets (inner need, recognition of a Creator) are hypothesized to relate to cooperative conflict-resolution styles (H4a); and the extrinsic facet (attention to external marks) to authoritarian management style (H4b), with H4 predicting stronger associations for the intrinsic than the extrinsic paths to positive managerial behaviors. Gender, age, and managerial level enter the model as adjustment covariates, and H5 concerns gender differences in religiosity and conflict-handling style. No mediation or moderation effects are hypothesized, accordingly, the analytic strategy (Section 3.5) tests direct associations with demographic adjustment rather than structural or conditional process models.

## 3 Materials and methods

### 3.1 Design

The design was correlational and cross-sectional. Data were collected during March–April 2026 using an anonymous online self-report questionnaire that was distributed through social-media platforms.

### 3.2 Participants

Participants were recruited from a self-selected (convenience) online sample (Table 1). Recruitment links were posted on social-media platforms targeting managerial and supervisory professionals in Uzbekistan. Participation was voluntary, anonymous, and unpaid. Sampling was conducted in a single stage: one open recruitment link was distributed, and every eligible respondent who followed the link and submitted the questionnaire was retained. No clusters, strata, or quotas were used at any point, and no second selection stage occurred; the procedure was therefore single-stage convenience sampling rather than two-stage or stratified sampling. Inclusion criteria were (a) current employment in a managerial or supervisory position in an organization operating in Uzbekistan, (b) age 18 years or older, and (c) sufficient proficiency in Uzbek to complete the instruments. The only exclusion criterion was an incomplete submission; because all psychometric items in the online form were mandatory, all 286 submitted cases contained complete scale data and none was excluded. Twelve respondents (4.2%) did not report their managerial level; these cases were retained and are omitted only from analyses that include managerial level as a covariate, which accounts for the reduced degrees of freedom in the corresponding regression models reported in Section 4.4. A total of 286 participants (211 male, 73.8%; 75 female, 26.2%) occupying entry-level, middle, or senior management positions in education, private sector enterprises, and state organizations in Tashkent and several regional centers in Uzbekistan. The achieved sample was distributed across three sectors (education *n* = 99, 34.6%; private *n* = 97, 33.9%; state *n* = 90, 31.5%) and three managerial levels (entry *n* = 163, 57.0%; middle *n* = 45, 15.7%; senior *n* = 66, 23.1%). Age was treated as a categorical variable (under 30: *n* = 73, 25.5%; 30–40: 74, 25.9%; 40–50: *n* = 93, 32.5%; over 50: *n* = 46, 16.1%). These proportions are representative of the achieved sample, not pre-set quotas, as recruitment was via open online distribution and not via controlled quota recruitment. The predominance of men is generally in line with the gender imbalance in Uzbek managerial positions, where men are reported to hold between 60% and 85% of high and mid-level management jobs, depending on the sector (UNFPA, 2025).

**Table 1:** Sociodemographic characteristics of the sample (*n* = 286).

Variable	Category	*N*	%
Gender	Male	211	73.8
	Female	75	26.2
Age	Under 30	73	25.5
	30–40	74	25.9
	40–50	93	32.5
	Over 50	46	16.1
Sector	Education	99	34.6
	Private	97	33.9
	State	90	31.5
Managerial level	Entry	163	57
	Middle	45	15.7
	Senior	66	23.1

### 3.3 Measures

#### 3.3.1 Religiosity

##### 3.3.1.1 Santa clara strength of religious faith questionnaire (SCSORF)

The 10-item SCSORF (Plante, 2020; Plante and Boccaccini, 1997a; Plante et al., 2002) is a brief measure of strength of religious faith, a four-point response format that is denominationally neutral. The scale was administered in the Uzbek translation. Its reliability and validity were established in the original development studies (Plante and Boccaccini, 1997a,b) and have since been supported across numerous languages, cultures, and faith traditions (Plante, 2021) of particular relevance to the present setting, the scale has been validated in a Muslim sample (Pakpour et al., 2014), supporting its use with Uzbek Muslim respondents. The current sample showed good internal consistency (α = 0.93). A Kaiser-Meyer-Olkin (KMO) value of 0.91 and significant Bartlett's test of sphericity [χ^2^_(45)_ = 1,833.18, *p* < 0.001] confirmed the unidimensional factor structure.

##### 3.3.1.2 Shcherbatykh personal structure of religiosity

Developed by Myagkov et al. (1996) and widely used in Russian-language psychology, this 40 item scale measure eight dimensions of religiosity with a 5-point response format: (a) philosophical orientation toward religion; (b) belief in the mystical world; (c) religion as a source of consolation; (d) attention to external religious markers; (e) inclination toward pseudoscience; (f) recognition of a Creator; (g) inner religious need; and (h) view of religion as a moral norm. A composite score for “general religiosity” was created from six positively valenced subscales (α = 0.87). The KMO value was 0.89, and Bartlett's test was significant [χ^2^_(780)_ = 4,455.74, *p* < 0.001]. Subscale reliabilities ranged from α = 0.14 (Philosophical) to α = 0.72 (Inner Need). Subscales of acceptable reliability (inner need, recognition of a Creator) and the general-religiosity composite are given interpretive weight. Subscales of low reliability are treated as exploratory and discussed under Limitations.

#### 3.3.2 Management styles

Ilin Management Style Inventory (abridged 33-item version). An inventory of 33 items measuring authoritarian, democratic, and liberal styles scored 0 to 11 on each style. The highest score was used as the dominant style for each participant. The inventory is designated here by the name under which it circulates in the post-Soviet literature. Its item pool derives from the abridged 33-item form of Self-Assessment of Management Style questionnaire published by Agrashenkov (1997), and the administered in the present study is the version reproduced in Il'in's compendia of differential and occupational psychology Il'in, (2008). Internal consistency was acceptable for the liberal subscale (α = 0.69) but low for authoritarian (α = 0.45) and democratic (α = 0.33) subscales. Since Cronbach's α is sensitive to the number of items and the heterogeneity of the inter-item correlations (Cortina, 1993), these two subscales are treated as exploratory in Limitations, and interpreted with caution. The study's core findings do not rely on them.

#### 3.3.3 Self-regulation

Nikiforov Integral Self-Control Scale. A 36-item scale with three subscales (emotional, performance, and social self-control) and a composite overall self-control score (α = 0.62 for the composite). The questionnaire was developed by Nikiforov, Vasil'ev, and Firsova on the basis of Nikiforov's (1989) monograph on human self-control, in which self-control is conceptualized as the establishment of correspondence between a person's activity and psychological processes, on one side, and the norms and requirements placed upon that person, on the other. In the post-Soviet psychological literature the instrument is widely used.

#### 3.3.4 Style of the conflict resolution

Thomas-Kilmann Conflict Mode Instrument (TKI). The 30-item ipsative TKI (Thomas and Kilmann, 1974) measures five conflict management styles: competing, collaborating, compromising, avoiding, and accommodating. The instrument's forced-choice format was developed and validated by Kilmann and Thomas (1977), who constructed the item pairs to be matched on social desirability; comparative psychometric evidence against other conflict-behavior measures is reported in Thomas and Kilmann (1978). Because the instrument is ipsative (forced-choice), Cronbach's α is not a suitable index of reliability.

### 3.4 Procedures and ethical considerations

Implied informed consent was obtained from all participants, as the completion of the anonymous voluntary survey was considered consent. The study was conducted in accordance with the ethical principles of the Declaration of Helsinki and APA standards. Anonymity was assured, and no personally identifying information was gathered. It was a voluntary and unpaid activity. Ethical approval: it was taken from the Department of Psychology of Religion and Pedagogy at the International Islamic Academy of Uzbekistan and the Committee on Religious Affairs under the Cabinet of Ministers of the Republic of Uzbekistan.

### 3.5 Statistical analyses

All statistical analyses were performed using IBM SPSS Statistics 26.0. Descriptive statistics (means, standard deviations, skewness, and kurtosis) were calculated for all variables. Internal consistency was assessed by Cronbach's α. Factorability was assessed by the KMO statistic and Bartlett's test of sphericity. Pearson product-moment correlations were performed to examine bivariate associations, alongside parallel tests with Spearman's ρ. Group differences were tested with one-way ANOVA (*post-hoc* comparisons with Tukey's HSD), independent samples *t*-tests with Welch's correction where appropriate, and χ^2^ tests of independence with Cramer's *V*. Multivariate predictions were assessed with linear regression entered with religiosity indices and demographic covariates (gender, age, and managerial level); multicollinearity was evaluated by the variance inflation factor (all VIF < 2.5). Effect sizes (*r*, η^2^, and Cohen's *d*) were interpreted using both Cohen's (1988) classical benchmarks and the more recent recommendations by Funder and Ozer (2019). The level of significance was set at α = 0.05, and results are reported in accordance with APA reporting standards (American Psychological Association, 2020).

The analytic strategy was selected to match the study's aims, which were associational rather than structural. Because no quantitative evidence on religiosity and managerial behavior existed for this population, the purpose was first to establish whether, and for which dimensions, associations exist. Bivariate correlations were therefore computed for transparency and comparability with the prior literature, in which effects are conventionally reported in the correlation metric (McCullough and Willoughby, 2009), and multivariate linear regression was then used to test whether these associations survive adjustment for demographic covariates—a stronger test than bivariate analysis alone. More complex techniques such as structural equation modeling or conditional-process (mediation/moderation) analysis were deliberately not employed, for two reasons. First, the research model (Figure 1) advances only direct-association hypotheses, so a structural model would test claims the study does not make. Second, the modest internal consistency of several subscales and the ratio of sample size to the number of observed indicators would render latent-variable estimates unstable (Hair et al., 2010; West et al., 1995). The rationale is thus explicit: the analyses were designed solely to establish and demographically adjust associations, and structural modeling is identified under Limitations as a task for larger, better-instrumented samples.

## 4 Results

### 4.1 Psychometric properties and descriptive statistics

Descriptive statistics for main variables are presented in Table 2. The mean SCSORF score of the sample was 32.07 (SD = 7.52) or 80.2% of the maximum possible score on the scale. Seventy-one percent of the sample scored in the high belief band (≥30), 21.3% in the moderate band (20–29), and 7.7% in the low band ( ≤ 19). The most strongly endorsed Shcherbatykh dimensions were the Inner Need (*M* = 7.52), Attention to External Markers (*M* = 7.16), and Recognition of a Creator (*M* = 6.71), whereas Pseudo-science (*M* = 3.86) was the least endorsed.

**Table 2:** Descriptive statistics for the principal study variables (*n* = 286).

Scale	Range	*M*	SD	Skew.	**Kurt**.
SCSORF (religious faith)	10–40	32.07	7.52	−1.15	0.74
Sh-Philosophical orientation	0–10	6.19	1.54	−0.66	1.75
Sh-Mystical world	0–10	5.32	2.13	−0.30	0.01
Sh-Source of consolation	0–10	5.08	1.66	−0.48	0.92
Sh-External markers	0–10	7.16	2.29	−0.95	0.47
Sh-Pseudo-science	0–10	3.86	2.15	0.27	−0.39
Sh-Recognition of Creator	0–10	6.71	1.60	−1.53	3.83
Sh-Inner Need	0–10	7.52	2.16	−1.26	1.52
Sh-Moral norm	0–10	6.37	1.82	−0.54	0.59
Sh-General religiosity (6 positive)	0–60	39.13	8.12	−0.73	0.71
Authoritarian style (Ilin)	0–11	5.93	1.82	0.44	0.25
Democratic style (Ilin)	0–11	8.38	1.43	−0.51	0.53
Liberal style (Ilin)	0–11	5.54	2.47	0.21	−0.79
Emotional self-control	–	12.92	2.29	−0.31	−0.36
Performance self-control	–	14.23	1.86	−0.86	0.28
Social self-control	–	16.48	2.00	−0.29	−0.03
Integral self-control	–	43.63	4.47	−0.46	−0.03
TKI-Competing	0–12	7.43	1.53	−0.16	−0.45
TKI-Collaborating	0–12	5.45	1.56	0.24	−0.28
TKI-Compromising	0–12	5.26	1.69	0.10	−0.37
TKI-Avoiding	0–12	4.31	1.55	0.32	0.23
TKI-Accommodating	0–12	7.55	1.86	−0.03	0.01

Skewness and kurtosis are within the range (skew. < 2; kurt. < 7) that permits analysis (West, Finch, and Curran 1995).

The dominant management style was democratic for 202 respondents (70.6%), liberal for 25 (8.7%), authoritarian for 16 (5.6%), and mixed profiles for 43 (15.0%). Conflict-handling dominant style was competing 139 (48.6%), accommodating 98 (34.3%), compromising 25 (8.7%), collaborating 20 (7.0%), and avoiding 4 (1.4%).

### 4.2 Bivariate relationships between religiosity and managerial behavior

Pearson and Spearman correlations were performed between the nine religiosity variables and the twelve managerial behavior variables, producing 108 pairs tested. Table 3 shows the statistically significant associations. The main positive findings were that the total score of the SCSORF predicted the accommodating conflict style (*r* = +0.17, *p* = 0.004), and on the dimensional level, the subscales Shcherbatykh's Inner Need and Recognition of a Creator were the strongest predictors of accommodation (*r* = +0.17, *r* = +0.17, and *p*s < 0.01). Similarly, the collaborating style was negatively correlated with the same two intrinsic religiosity subscales (*r* = −0.17, *r* = −0.12, and *p*s < 0.05).

**Table 3:** Statistically significant correlations between religiosity variables and managerial behavior variables (*n* = 286).

Religiosity variable	Outcome	*r* (Pearson)	*p*	ρ (Spearman)	*p*
SCSORF	TKI-accommodating	+0.17^**^	0.004	+0.16^**^	0.007
Sh-philosophical	Performance self-control	−0.12^*^	0.038	−0.13^*^	0.030
Sh-consolation	Performance self-control	−0.14^*^	0.019	−0.12^*^	0.036
Sh-creator	Performance self-control	−0.07	0.216	−0.12^*^	0.046
Sh-creator	TKI-collaborating	−0.17^**^	0.004	−0.17^**^	0.003
Sh-creator	TKI-accommodating	+0.17^**^	0.003	+0.19^**^	0.001
Sh-inner need	TKI-collaborating	−0.12^*^	0.039	−0.11	0.063
Sh-inner need	TKI-accommodating	+0.17^**^	0.004	+0.16^**^	0.009

^*^p < 0.05, ^**^p < 0.01. Only statistically significant pairs from 108 tested are shown.

Of particular note, the SCSORF total was not significantly associated with democratic style (*r* = −0.03, *p* = 0.635) or with integral self-control (*r* = −0.03, *p* = 0.663), failing to support H1 and H2 in their direct form.

### 4.3 ANOVA: religious belief groups and conflict-handling styles

A one-way ANOVA analysis showed that there were statistically significant differences among the three SCSORF groups (low *n* = 22, moderate *n* = 61, and high *n* = 203) on two TKI styles: Competing [*F*_(2, 283)_ = 4.14, *p* = 0.017, and η^2^ = 0.028] and Accommodating [*F*_(2, 283)_ = 4.74, *p* = 0.009, and η^2^ = 0.032]. No significant differences in management style and self-control indices were found between groups (all *p* > 0.05). Tukey's HSD *post-hoc* tests revealed that low-belief participants scored significantly higher on competing than moderate-belief (M difference = +0.99, *p* = 0.024) and high-belief participants (M difference = +0.95, *p* = 0.015), and high-belief participants scored significantly higher on accommodating than the other groups (Table 4).

**Table 4:** Results of ANOVA for religious belief groups on managerial behavior variables (*n* = 286).

Outcome	Low *M* (SD)	Moderate *M* (SD)	High *M* (SD)	*F*_(2, 283)_	*p*	η^2^
Authoritarian	6.77 (2.33)	6.38 (1.80)	5.76 (1.75)	2.67	0.071	0.019
Democratic	8.68 (1.76)	8.30 (1.46)	8.40 (1.40)	0.34	0.712	0.002
Liberal	5.91 (2.93)	5.59 (2.35)	5.48 (2.46)	0.31	0.733	0.002
Integral self-control	43.86 (4.84)	43.84 (3.68)	43.54 (4.66)	0.14	0.872	0.001
TKI-competing	8.32 (1.81)	7.33 (1.45)	7.36 (1.49)	4.14	0.017^*^	0.028
TKI-collaborating	5.82 (1.56)	5.54 (1.57)	5.38 (1.55)	0.90	0.408	0.006
TKI-compromising	4.82 (1.18)	5.62 (1.70)	5.20 (1.72)	2.29	0.103	0.016
TKI-avoiding	4.09 (1.23)	4.46 (1.52)	4.29 (1.59)	0.52	0.597	0.004
TKI-accommodating	6.95 (2.15)	7.05 (1.67)	7.76 (1.85)	4.74	0.009^**^	0.032

^*^p < 0.05, ^**^p < 0.01. η^2^ interpretation: 0.01 = small, 0.06 = medium, and 0.14 = large (Cohen, 1988).

### 4.4 Multivariate regression models

Multivariate linear regression models were fitted for each managerial behavior outcome to examine the joint contribution of religiosity and demographic covariates. Table 5 shows the results for the four most informative models.

**Table 5:** Multivariate linear regression models to predict managers' behavior variables.

Outcome/predictor	*B*	SE	β	*t*	*p*
TKI-Accommodating [***R***^2^ = **0.077**, *F*_(6, 208)_ = **2.90, and** ***p*** = **0.010]**					
SCSORF	0.021	0.022	0.090	0.95	0.341
Sh-inner need	−0.001	0.081	−0.001	−0.01	0.994
Sh-recognition of creator	0.091	0.100	0.084	0.91	0.363
Gender (1 = male)	−0.068	0.295	−0.016	−0.23	0.820
Age (categorical)	0.445	0.153	0.238	2.91	0.004^**^
**TKI-Collaborating [*****R***^2^ = **0.068**, *F*_(5, 280)_ = **4.11, and** ***p*** = **0.001]**					
SCSORF	−0.011	0.017	−0.051	−0.64	0.524
Sh-inner need	0.008	0.063	0.011	0.13	0.899
Sh-recognition of creator	−0.140	0.079	−0.144	−1.78	0.075^†^
Gender (1 = male)	−0.666	0.209	−0.189	−3.19	0.002^**^
Age	−0.061	0.089	−0.040	−0.69	0.493
**Authoritarian [*****R***^2^ = **0.040**, *F*_(5, 280)_ = **2.35, and** ***p*** = **0.041]**					
SCSORF	−0.028	0.020	−0.115	−1.38	0.169
Sh-inner need	−0.077	0.074	−0.090	−1.04	0.297
Sh-external markers	0.129	0.066	0.163	1.96	0.051^†^
Gender (1 = male)	−0.409	0.251	−0.099	−1.63	0.103
Age	−0.126	0.106	−0.072	−1.19	0.234
**Democratic [*****R***^2^ = **0.035**, *F*_(6, 208)_ = **1.27, and** ***p*** = **0.272]**					
SCSORF	−0.007	0.017	−0.040	−0.42	0.673
Sh-inner need	0.007	0.064	0.012	0.11	0.909
Sh-moral norm	0.071	0.060	0.101	1.18	0.240
Gender (1 = male)	−0.188	0.224	−0.059	−0.84	0.403
Age	0.181	0.115	0.131	1.57	0.118

^†^*p* < 0.10, ^**^p < 0.01. All VIF < 2.5.

Two findings deserve emphasis. First, the bivariate religiosity-accommodation association did not survive multivariate adjustment for age; age itself emerged as the dominant predictor (β = 0.24, *p* = 0.004). This indicates substantial shared variance between religiosity and age in the sample—older managers report both stronger faith and more accommodating styles. Second, the extrinsic religiosity facet (attention to external markers) was a marginal positive predictor (β = 0.16, *p* = 0.051) in the authoritarian model, and no intrinsic facet predicted authoritarianism. While non-significant at the traditional threshold, this trend is in the theoretically predicted direction and deserves testing in adequately powered replications.

### 4.5 Categorical independence tests

χ^2^ tests treating dominant management style and dominant conflict style as categorical outcomes found no significant association with religious belief group (Table 6). This suggests that dimensional analyses are more sensitive than categorical analyses to detect religiosity effects in this sample.

**Table 6:** χ^2^ tests of independence.

**Test**	χ^2^	df	*p*	Cramer's *V*
Dominant management style × belief group	7.28	6	0.296	0.113
Dominant conflict style × belief group	11.06	8	0.198	0.139

### 4.6 Gender differences

Independent-sample *t*-tests revealed several substantive gender effects (Table 7). Women scored higher on SCSORF (*d* = −0.26, *p* = 0.041), the liberal management style (*d* = −0.34, *p* = 0.013), and the collaborating conflict style (*d* = −0.44, *p* = 0.001). Men scored higher on the compromising style (*d* = +0.33, *p* = 0.012). No significant gender differences emerged for the democratic management style or for integral self-control.

**Table 7:** Gender differences in principal variables.

Variable	Male *M* (SD)	Female *M* (SD)	*t*	*p*	Cohen's *d*
Authoritarian	5.80 (1.79)	6.29 (1.89)	−1.96	0.052	−0.27
Democratic	8.38 (1.43)	8.37 (1.41)	0.03	0.976	0.00
Liberal	5.32 (2.43)	6.16 (2.49)	−2.53	0.013^*^	−0.34
Emotional self-control	13.05 (2.31)	12.55 (2.20)	1.67	0.097	0.22
Integral self-control	43.95 (4.18)	42.71 (5.14)	1.89	0.061	0.28
TKI-collaborating	5.27 (1.57)	5.95 (1.42)	−3.42	0.001^***^	−0.44
TKI-compromising	5.41 (1.71)	4.85 (1.57)	2.56	0.012^*^	0.33
SCSORF (religious faith)	31.57 (7.76)	33.49 (6.65)	−2.06	0.041^*^	−0.26

^*^p < 0.05, ^***^p < 0.001.

### 4.7 Age variations

One way ANOVA across the four age groups showed significant linear age effect on TKI-Accommodating [*F*_(3, 282)_ = 5.72, *p* = 0.001], with mean scores increasing from 6.82 in the youngest group to 8.15 in the oldest. No other significant age effects on managerial behavior were found.

## 5 Discussion

### 5.1 Summary of results

We examined whether managerial behavior is predicted by religious belief in a Muslim-majority post-Soviet workforce and whether Allport's intrinsic–extrinsic distinction is empirically supported in this context. The results can be summed up in five points. First, religious belief is widespread and high in this Uzbek managerial sample (mean SCSORF 80% of maximum; 71% in the high-belief band), and the dominant management style is democratic (70.6%), and the dominant conflict-handling style is competing (48.6%), followed by accommodating (34.3%). Second, religious belief is reliably related to the accommodating conflict style and inversely related to competing but is not directly related to management-style preference (authoritarian/democratic/liberal) or to self-regulation (at the bivariate level). Third, the dimensional structure of religiosity is of relevance: intrinsic facets (inner need, recognition of a Creator) are responsible for the religiosity–accommodation relationship, whereas extrinsic religiosity (attention to external markers) is the only facet showing even a marginal (non-significant) trend toward authoritarianism. Fourth, the demographic predictors (age and gender) generally predict managerial behavior better than religiosity. The bivariate relationship between religiosity and accommodation drops to non-significance when age is added to the model. Fifth, categorical analyses were less sensitive to these effects than dimensional analyses.

### 5.2 Hypothesis-by-hypothesis evaluation

H1: Religious belief correlates positively with democratic and negatively with authoritarian management style. Not supported. Religious belief was unrelated to democratic style at the bivariate level (*r* = −0.03, *p* = 0.635), belief groups did not differ on any management style in the ANOVA (all *p* > 0.05), and no religiosity index significantly predicted democratic or authoritarian style in the regression models (Table 5). Two features of the sample plausibly contribute to this null result. First, the democratic style was near-universal (70.6% dominant; *M* = 8.38 of 11), so restricted variance limited the detectable association. Second, the low internal consistency of the authoritarian and democratic subscales constrained measurement precision. Substantively, the finding qualifies the assumption in values-based leadership scholarship that religious values translate directly into participative leadership (Brown et al., 2005; Fry, 2003): in a context where democratic self-presentation is normative, religiosity adds no incremental prediction of style preference.

H2: Religious belief is positively related to self-regulation. Not supported. Religious belief was not associated with integral self-control (*r* = −0.03, *p* = 0.663), seemingly at odds with McCullough and Willoughby's (2009) meta-analytic conclusion. Several explanations are plausible. The Nikiforov instrument measures self-control in a behavior-focused, organizationally framed way that may tap different content than the moral and impulse-control measures aggregated in the meta-analysis. Some Nikiforov subscales showed low internal consistency (α = 0.35–0.52), restricting the variance available to detect correlations. And in a sample where 71% of participants fall in the high-belief band, the restricted range of belief may further reduce statistical power. Future work should test the religion–self-control link in this region using broader-bandwidth instruments and samples that include a meaningful number of low-belief participants.

H3: Religious belief correlates positively with cooperative conflict-resolution styles and negatively with competing. Partially supported, belief was positively associated with accommodating (*r* = +0.17, *p* = 0.004), and low-belief participants scored significantly higher on competing than moderate- and high-belief participants in the ANOVA. However, the second cooperative style, collaborating, was negatively—not positively—correlated with the intrinsic facets, and the bivariate belief–accommodation association attenuated to non-significance once age entered the regression model, with age emerging as the dominant predictor (β = 0.24, *p* = 0.004). The supported portion parallels the Islamic ethical emphasis on sabr (patience), tawakkul (trust in God), and forbearance as the proper posture toward interpersonal friction (Caputo, 2018; Wilson and Power, 2004) and is consistent with the collectivist preference for accommodation documented cross-culturally (Cai and Fink, 2002; Holt and DeVore, 2005). The unsupported portion is theoretically informative: collaboration in the Thomas–Kilmann framework requires high assertiveness as well as high cooperativeness, and managers whose religious commitment emphasizes self-restraint may experience the assertiveness pole of collaboration as inconsistent with their value commitments, even when the cooperative pole is congenial. The age finding indicates substantial shared variance between religiosity and age—older managers report both stronger faith and more accommodating styles—and cautions against interpreting the bivariate association causally.

H4: Intrinsic religiosity is more strongly associated with positive managerial behaviors than extrinsic religiosity. Partially supported, in the form of a differentiated pattern. The intrinsic facets (inner need, recognition of a Creator) were the religiosity variables associated with accommodating conflict handling, whereas the extrinsic facet (attention to external markers) was unrelated to any cooperative outcome and instead showed a marginal, non-significant positive trend toward authoritarian style (β = 0.16, *p* = 0.051)—the theoretically predicted direction, given the long line of evidence linking extrinsic orientation to conformity, conservatism, and authoritarian attitudes (Allport and Ross, 1967; Hill and Pargament, 2003; Saroglou, 2011). Because this trend does not reach conventional significance, it should be treated as a hypothesis for adequately powered replication rather than a confirmed effect. If replicated, it would represent one of the first demonstrations of this association in a Central Asian sample and would extend Saroglou's cross-cultural framework to a historically underrepresented region. Overall, the small size of the observed effects (*r* = 0.12–0.19; η^2^ = 0.028–0.032) does not negate their theoretical interest: by the effect-size framework of Funder and Ozer (2019), correlations in this range represent meaningful population-level tendencies, and they fall within the range McCullough and Willoughby (2009) identify as typical for this literature. They should nonetheless be understood as modest associations within a multivariate model in which demographic factors carry greater weight.

H5: Women are more religious than men and prefer more cooperative and accommodating conflict styles. Partially supported. Women scored higher on religious faith (*d* = −0.26, *p* = 0.041) and on the cooperative style of collaborating (*d* = −0.44, *p* = 0.001), consistent with cross-cultural meta-analyses of gender and religiosity (Francis and Penny, 2014) and of gender and leadership (Eagly and Carli, 2003; Eagly et al., 1995); women were also higher on liberal management style (*d* = −0.34). Contrary to prediction, however, no significant gender difference emerged on accommodating, and men scored higher on compromising (*d* = +0.33). That gender, not religiosity, was the strongest predictor of collaborating (β = −0.19, *p* = 0.002) underscores the importance of treating religiosity as one variable within a multivariate model of managerial behavior rather than as a stand-alone predictor.

### 5.3 Theoretical integration: spiritual and value-based leadership

Read through the lens of the study's guiding frameworks, the pattern of results is informative in three ways. First, spiritual leadership theory (Fry, 2003; Fry et al., 2017) locates the behavioral force of religion in internalized spiritual values—calling and membership—rather than in outward religious performance. The present findings support that premise at the level of individual differences: it was precisely the internalized facets of religiosity (inner need, recognition of a Creator), and not attention to external markers, that related to conflict-handling behavior. Second, however, the null results for H1 and H2 refine the theory's scope: internalized religiosity did not translate into democratic style or higher self-regulation in this sample, suggesting boundary conditions—for instance, that in a workforce where religious commitment and democratic self-presentation are both normative, spiritual values differentiate managers' conflict posture more than their declared leadership style. Third, the directional (non-significant) extrinsic–authoritarian trend is consistent with the social-learning logic of ethical and value-based leadership theory (Brown et al., 2005): where religious observance functions instrumentally rather than as an internalized moral standard, it supplies no ethical counterweight to directive, control-oriented management. The empirical evidence thus supports the core motivational premise of spiritual leadership theory, extends it to a post-Soviet Muslim-majority setting, and simultaneously contradicts any strong reading on which religious values uniformly produce participative leadership—a refinement that future theory-building should accommodate.

### 5.4 The methodological lesson: dimensional vs. categorical analyses

The χ^2^ tests using dominant style as a categorical outcome found no significant association with belief group, whereas dimensional analyses revealed several meaningful effects. This pattern is well-known in the cross-cultural psychology of religion (Hood et al., 2018) and highlights the need to preserve dimensional information when testing religiosity effects, especially when sample sizes are modest and effect sizes small.

### 5.5 Limitations

There are several limitations to be mentioned. First, participants were recruited via an anonymous online convenience sample distributed through social media, rather than via probability or controlled quota sampling. This self-selection process limits the representativeness of the sample and may bias it toward more digitally engaged and potentially more religiously expressive managers. Therefore, findings should be generalized with caution. Second, the cross-sectional design prevents causal inference; longitudinal and experimental designs are needed to test for temporal precedence and directionality. Third, the sample is from one Muslim-majority country, so the findings cannot be readily generalized to other religious or cultural contexts. Fourth, all measures are self-report and thus subject to common-method bias and social-desirability effects, especially where religious commitment is a valued trait in the public domain (Field, 2018), so the findings of leadership style are best understood as self-perceived preferences and not observed behaviors. Fifth, some subscales of the Ilin and Nikiforov instruments showed low Cronbach's α values (0.33–0.52). Low α on short scales does not necessarily invalidate construct validity but limits measurement precision (Cortina, 1993; Hair et al., 2010), and a programmatic effort to validate Uzbek-language adaptations is needed. Sixth, small effect sizes mean that religiosity explains a small portion of variance (around 1.5%−3.5%) in managerial behavior. Seventh, the lack of a qualitative element prevented the researchers from investigating the subjective experience of faith–work integration. Future research is advised to employ mixed-methods designs. Finally, because the design advanced only direct-association hypotheses, no structural, mediation, or moderation models were estimated; larger samples with more reliable Uzbek-language instruments would permit latent-variable structural modeling of the religiosity–behavior paths.

### 5.6 Theoretical and practical implications

From the perspective of organizational psychology, this study has three contributions. First, it shows that religious orientation is a meaningful difference predictor of managerial behavior in Central Asia, extending the cross-cultural reach of organizational behavior research into a largely missing Muslim-majority, post-Soviet context in the empirical literature. Second, it demonstrates that it is the motivational quality of religious belief rather than the intensity that determines which organizational behaviors are predicted. The results indicate that intrinsic orientation is selectively related to conflict-handling style, and extrinsic orientation is directionally (non-significantly) related to leadership authoritarianism. This finding speaks directly to debates in values-based leadership theory (Fry, 2003; Brown et al., 2005) about whether religion-related constructs have consistent effects across organizational outcomes or operate in differentiated and theoretically consistent ways. Third, the study suggests that demographic variables (age, gender) are generally better predictors of managerial behavior than religiosity. This should warn against giving too much weight to religious orientation in applied organizational models, while confirming that religiosity adds incremental variance consistent with Allport's (1950) account of religion as a motivational structure (Allport and Ross, 1967; Saroglou, 2011).

The findings suggest several considerations in practice for management education and HR policy in the region. First, religiosity should be seen as part of a wider value architecture interacting with age, gender, and professional experience rather than as a standalone predictor. Second, the well-documented tendency of high-religiosity managers to default to accommodation in conflict may be worthy of attention in management development programs, particularly where collaborative integration is the more organizationally optimal response (de Wit et al., 2012). Third, the marginal association of extrinsic religiosity with authoritarianism deserves attention in religious-educational and managerial-training programs aimed at developing reflective intrinsically motivated faith.

## 6 Conclusions

The study presents initial cross-sectional quantitative evidence from a Central Asian Muslim managerial workforce that Allport's intrinsic–extrinsic distinction in religiosity is associated with differentiated organizational behavioral correlates. In this convenience sample of 286 Uzbek managers, the dominant leadership style was democratic (70.6%) and the dominant conflict handling mode was competing (48.6%), followed by accommodating (34.3%). Religious belief was consistently associated with conflict handling accommodation at the bivariate level, but this association was driven by facets of intrinsic religiosity (inner need and recognition of a Creator) and was attenuated to non-significance once age was included as a covariate. Only the extrinsic facet (attention to external markers) showed a marginal, not significant, tendency toward authoritarian leadership style. Religiosity was not a direct predictor of either leadership style preference or self-regulation, and demographic variables (age, gender) tended to explain more variance than religiosity. These findings extend cross-cultural organizational psychology in three ways. First, they extend individual-difference research on managerial behavior to a Muslim-majority, post-Soviet context. Second, they provide preliminary evidence for the organizational relevance of the intrinsic–extrinsic distinction in a non-Western, non-Christian sample. Third, they provide an empirical baseline for future longitudinal, experimental, and mixed-method research on religion, leadership, and workplace conflict in Central Asia.

## Data Availability

The datasets presented in this study can be found in online repositories. The names of the repository/repositories and accession number(s) can be found below: https://osf.io/bygq8.
